# Malignant Extra-Gastrointestinal Stromal Tumor of the Mesentery

**DOI:** 10.1055/s-0039-1693040

**Published:** 2019-08-01

**Authors:** Prakash K. Sasmal, Rakesh Sharma, Susama Patra, Tushar S. Mishra, Pritinanda Mishra, Bikram Rout

**Affiliations:** 1 Department of General Surgery, All India Institute of Medical Sciences, Bhubaneswar, Odisha, India; 2 Department of Pathology, All India Institute of Medical Sciences, Bhubaneswar, Odisha, India

**Keywords:** malignant GISTs, mesentery, positron emission tomography scan, immunohistochemistry, imatinib mesylate

## Abstract

Gastrointestinal stromal tumors (GISTs), the commonest mesenchymal tumors of gastrointestinal tract are often described to take origin from the interstitial cells of Cajal (ICC) or its precursor cells. Rarely these tumors do arise in structures other than the alimentary tract like omentum, mesentery, retroperitoneum, etc., of varying malignant potential and are known as extra-gastrointestinal stromal tumors (eGISTs).

This is a case report of a 70-year-old female with multicentric malignant eGISTs arising in the mesentery of ileum. On laparotomy, a large mass of 20 × 15 cm was found in the small bowel mesentery without involvement of the adjacent ileum, with multiple other small nodules resembling lymph nodes, present adjacent to it. Histopathological study of the excised lump, confirmed the mass to be malignant eGIST without involvement of the adjacent ileum, with cluster differentiation (CD)117 positive and of high-risk stratification. The mesenteric nodule was confirmed on histopathology to be malignant eGIST, similar to that of that of the primary, without any lymphoid tissue. Adjuvant imatinib mesylate treatment was started immediately postoperation with the patient doing well at 1 year of follow-up. We report this case, due to the rare occurrence of multifocal malignant eGISTS of small bowel mesentery, which is yet to be reported. The existing literature is unclear regarding the clinicopathology and management of multifocal malignant stromal tumors of the mesentery.

Gastrointestinal stromal tumors (GISTs) are the most common mesenchymal tumors of gastrointestinal tract. However it comprises only less than 1% of all primary gastrointestinal malignancies. 1 Although they can arise anywhere in the gastrointestinal tract, stomach is more frequently involved (60–70%), followed by small intestine (25–35%), colon, rectum and appendix together (5%), and esophagus (2–3%). 2 Primary GISTs can rarely occur away from the bowel in the mesentery, omentum, retroperitoneum, etc., which are often referred to as extra-gastrointestinal stromal tumors (eGISTs). 3 Like the stromal tumors, eGISTs also are phenotypically related to the gastrointestinal pacemaker cells (the interstitial cells of Cajal), as most of them express c-kit receptor. eGISTs are supposed to have a more aggressive course like the small bowel GISTs, compared with those arising in the stomach. 3 Lymph node involvement is seldom reported. 4 The use of specific tyrosine kinase receptor inhibitors has shown promising response in the treatment of the disease. We report here an interesting case of multifocal malignant eGISTs arising in the small bowel mesentery in an elderly female. Our case was unique as the multifocal presentation of the malignant eGISTs was only confirmed in the postoperative period by histopathological evaluation of the suspected lymph nodes.

## Case Report

**Fig. 1:**
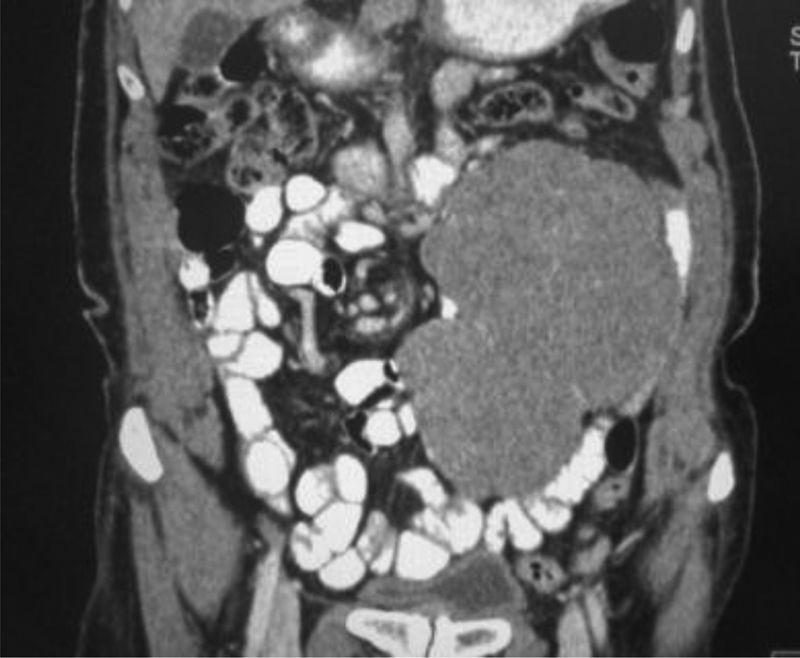
Contrast enhanced CT scan of abdomen showing the lump in the mesentery. CT, computed tomography.

A 70-year-old female presented to our hospital with a periumbilical abdominal lump associated with intermittent colicky abdominal pain. There was a rapid increase in size of the lump in the past few weeks. On evaluation, the abdominal examination revealed a well-defined, freely mobile, firm, intra-abdominal lump approximately 20 × 15 cm in size, occupying the left hypogastrium and paraumbilical region. Ultrasonography (USG) followed by contrast enhanced computed tomography (CECT) scan revealed a heterogeneous enhancing mass lesion, arising from the mesentery of small bowel, without any obvious involvement of the bowel ( Fig. 1 ).

**Fig. 2:**
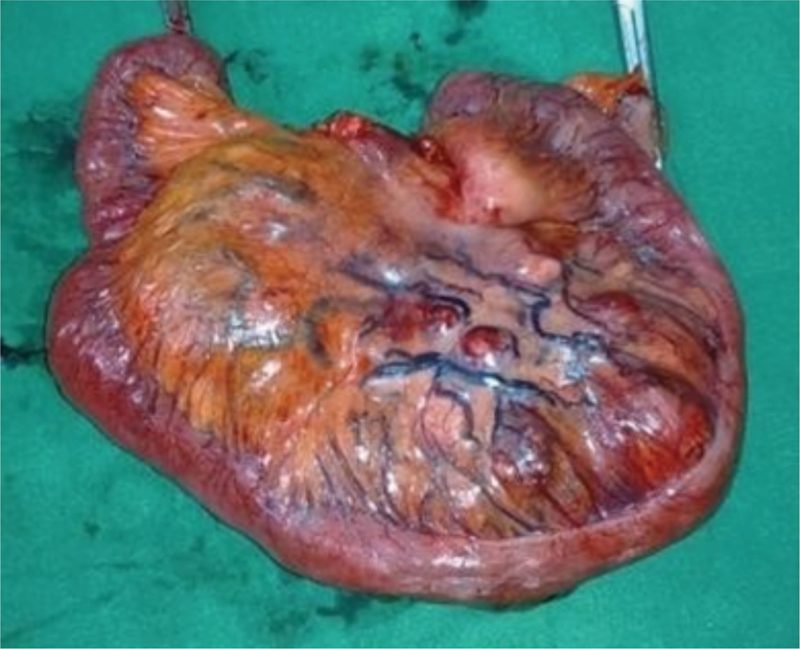
Excised specimen of eGIST in the mesentery with adjacent bowel. extra-gastrointestinal stromal tumors

**Fig. 3:**
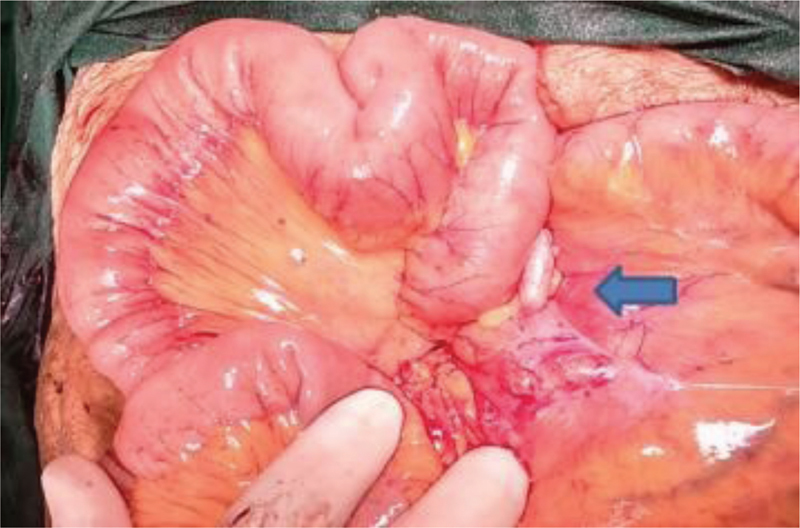
Multiple nodules (blue arrow) in the root of small bowel mesentery.

**Fig. 4:**
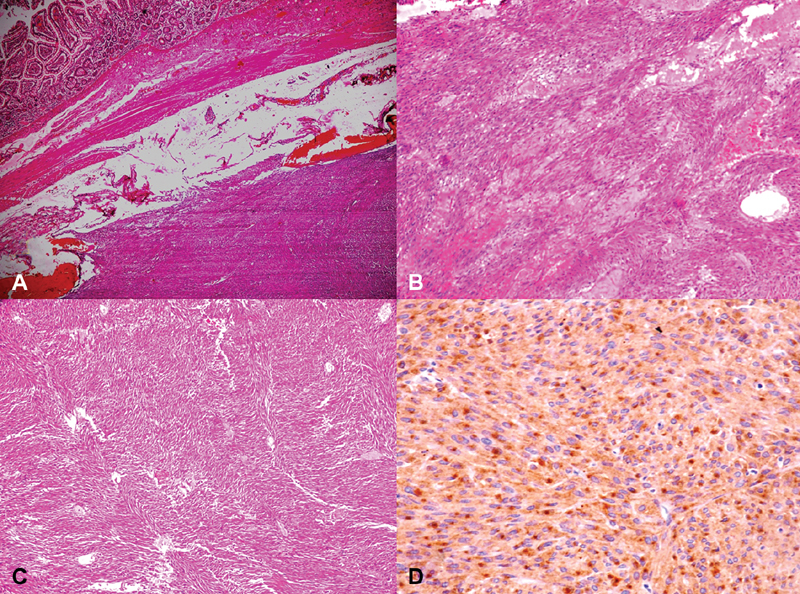
( **A** ) The tumor is arising from the mesentery with the overlying bowel wall free of involvement (H & E, ×40). ( **B** ) Tumor composed of long intersecting fascicles of spindle cells (H & E, ×100). ( **C** ) Myxoid areas with dilated vascular and congested channels (H & E, ×100). ( **D** ) Tumor cells showing paranuclear dot positivity for CD117 (IHC, ×100). H & E, hematoxylin and eosin; IHC, immunohistochemistry.

Exploratory laparotomy revealed a mobile, lobulated, highly vascular mass of variegated consistency in the mesoileum, about two feet proximal to ileocecal junction. The adjoining bowel was pushed to the periphery of the mass, without evidence of any gross involvement. A few other smaller nodules were present in the adjacent mesentery, near the mass, mimicking enlarged mesenteric lymph nodes without any evidence of involvement of other adjacent organs. En bloc resection of the mass along the mesenteric nodules and the adjacent small bowel was done followed by an end to end anastomosis ( Fig. 2 ). Multiple other smaller nodules in the adjacent mesentery of the small bowel were left behind assuming them to be enlarged lymph nodes ( Fig. 3 ). Histopathological examination of the tumor revealed long interlacing fascicles of malignant spindle cells with plump to cigar shaped blunt end nuclei with granular chromatin, moderate amount of eosinophilic granular cytoplasm, and ill-defined cytoplasmic border ( Fig. 4B and C ). Average mitotic rate was more than 5/50 high-power fields with large areas of necrosis, hemorrhage, and cystic degeneration. The overlying bowel wall was free from the tumor ( Fig. 4A ). The nodule which was sent separately assuming it to be lymph nodes was having same features of malignant eGIST without any lymphoid tissue. Immunohistochemical examination revealed the tumor to be strongly positive for cluster differentiation (CD)117, whereas negative for SMA (smooth muscle actin) and S-100, to rule out tumors with neural differentiation ( Fig. 4D ). Based on these findings a diagnosis of primary malignant eGIST of the mesentery of the high-risk category was made.

**Fig. 5:**
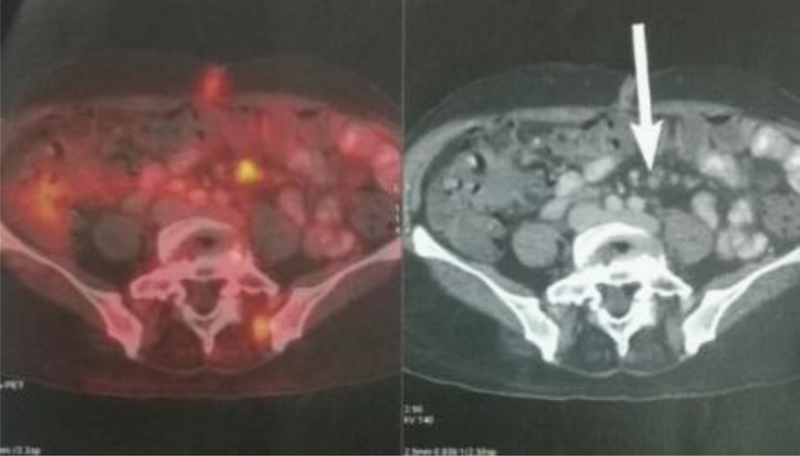
PET scan showing increased activity in the small bowel mesentery with multiple nodules seen in the computerized tomography scan (white arrow) indicating malignant eGISTs to be PET avid. eGIST, extra-gastrointestinal stromal tumors; PET, positron emission tomography.

Fluorodeoxyglucose positron emission tomography (FDG-PET) scan was done in the postoperative period to look for other sites of metastasis. There was increased activity in the tumor bed area, probably because of the residual nodules, left in the small bowel mesentery, which were intraoperatively assumed to be mesenteric lymph nodes ( Fig. 5 ). As the patient was elderly and frail, so was planned for adjuvant treatment. The patient was discharged on postoperative day 7 with imatinib mesylate (400 mg/day; Glivec, Novartis Pharma AG), as adjuvant chemotherapy and advice to follow-up at regular intervals. After 1 year of surgery, the patient is having a stable disease both clinically and on CECT scan.

## Discussion

GISTs are mesenchymal tumors arising from the interstitial cells of Cajal (ICC) or its precursor cells. The incidence varies from 0.1 to approximately 5% of all gastrointestinal tumors. 5 Tumors that are not connected to the gastrointestinal tract are known as extra-gastrointestinal stromal tumors (eGISTs) and constitute approximately 10% of all stromal tumors. 3 The common sites of involvement of eGISTs are omentum, mesentery, and retroperitoneum, although isolated cases of eGISTs at other locations, such as mesoappendix, seminal vesicles, urinary bladder, rectovaginal septum, pancreas, pleura, and prostate gland have been reported. 5 6 7 8 9 10 11 12 13 EGISTs have been postulated to be originating either from exophytic GISTs that arose within the gastrointestinal tract but eventually lost connection with it or primarily arising away from gastrointestinal tract. The predominant histological pattern is spindle-cell type (70%) which appears as fusiform cells in intersecting whorls. Other less common patterns seen are epithelioid types (20%) appearing as rounded cells in a nested pattern and rarely mixed pattern. 3 Both GISTs and eGISTs have the same histological, immunohistochemical behavior, as well as molecular biology with mutations in tyrosine-protein kinase (KIT) and platelet-derived growth factor receptor α (PDGFRA), which encodes a receptor tyrosine kinase that is strongly expressed in ICC. 14 Immunohistochemically strong positivity is noted for CD117 (> 95%) and CD34 (70%), whereas occasional positivity for smooth muscle actin (30%), S-100 (5%), desmin (2%), and cytokeratin (2%) have also been reported. 14

The symptoms associated with GISTs are largely depending upon the location and may cause gastrointestinal bleeding, abdominal pain, anemia, or abdominal mass, whereas smaller lesions may be incidentally detected during radiological evaluation or laparotomy. Anatomic location, size more than 10 cm, mitotic rate more than five per high-power field, and tumor rupture having the worse prognosis in terms of recurrence or metastasis. 15 Based on these criteria, our case falls into high-risk stratification. These tumors generally metastasize to liver or disseminate throughout peritoneal cavity but do not metastasize to lymph nodes (except for succinate dehydrogenase [SDH] deficient GISTs, a subset of GISTs with predilection for gastric location). 4 14 15 16 In our case, we found multiple mesenteric nodules adjacent to the main tumor mass spreading in the residual mesentery which had appearance of enlarged mesenteric lymph nodes. Histopathological examination, however, reported the nodules to be malignant eGISTs similar to the primary tumor without any lymphoid tissue. Omental GISTs have more favorable outcome compared with mesenteric GISTs. 17 Surgical resection with adjuvant specific tyrosine kinase inhibitors like imatinib mesylate therapy is the preferred treatment at present, inspite of high recurrence rate and development of resistance to it. 18 Sunitinib malate (SUTENT, SU11248, Pfizer Inc.), is efficacious in those cases showing resistance or partial response to imatinib. The GISTs have a high rate of recurrence depending on the risk stratification. In a study of 200 patients of malignant GISTs, about half of them had metastatic disease at presentation. 19 The median survival of the patients in the above study, with malignant GIST was approximately 19%. Hence, regular follow-up is essential to detect the recurrent disease early. The study for mutational analysis KIT exon-9 can predict the tumor aggressiveness and potential response to therapy. 20 In our case, with multifocal malignant eGISTs, which is yet to be reported, needs strict follow-up to study the disease course.

## Conclusion

Malignant eGISTs, although uncommon, are reported to occur at various extra-gastrointestinal sites, including the mesentery with variable clinical presentations. EGISTs may be considered as a differential diagnosis for large mobile mesenteric masses. The clinical course of the malignant eGISTs is not known, especially when multifocal, so active follow-up is mandatory. Specific tyrosine kinase inhibitor, like imatinib mesylate shows impressive response in high-risk disease.
